# GraPhAI: Neural Networks for Solving Centrosymmetric Crystal Structures

**DOI:** 10.1021/jacs.6c05607

**Published:** 2026-06-30

**Authors:** Džonatans Miks Melgalvis, Toms Rekis

**Affiliations:** 1 Faculty of Medicine and Life Sciences, 61769 University of Latvia, Jelgavas iela 1,LV1004 Riga,Latvia; 2 Institute for Inorganic and Analytical Chemistry, Goethe-University Frankfurt, Max-von-Laue Straße 7, 60438 Frankfurt am Main,Germany

## Abstract

Crystal structure determination from low-resolution diffraction data is challenging due to the lack of a general-purpose method for solving the crystallographic phase problem. We have developed a graph neural network, GraPhAI, and propose a new and efficient diffraction data representation in a graph form for deep learning. The trained GraPhAI models are intended for ab initio phasing of down to 2 Å resolution data of typical unit-cell volume centrosymmetric crystal structures. The success rate is above 80% for structures containing an atom with *Z* ≥ 19 (e.g., metal–organic frameworks, coordination compounds, or inorganic structures). For purely organic crystal structures, the success so far is limited.

## Graphical abstract

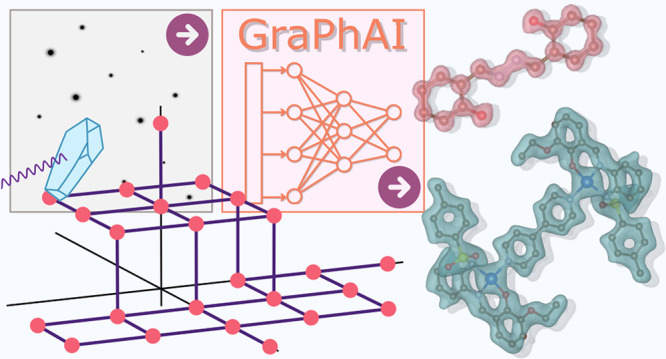

## Introduction

Recently, a breakthrough in ab initio crystal structure determination from low-resolution single-crystal diffraction data has been reported by tackling the crystallographic phase problem with deep learning.^1^ The neural network PhAI was successful in solving small unit cell crystal structures from as low as 2 Å resolution data in space group *P*2_1_/*c*. This proof-of-concept study has opened a new chapter in small-molecule crystallography since previously existing phasing methods are known to perform poorly with low-resolution data. Diffraction data completeness and resolution are generally low in electron and high-pressure diffraction due to the experimental setups^2,3^ and, in the case of the former, due to the nature of electron–matter interactions.^4^ Furthermore, some crystalline material classes, like metal–organic frameworks (MOFs), often produce weakly diffracting samples leading to low-resolution data even in the conventional single-crystal X-ray diffraction experiments.^5,6^ Further development in this field will enable structure determination from low-resolution and low-completeness data pushing the structural science forward.

Machine learning has been successfully exploited to tackle different problems in structural science, for example, aiding crystal structure determination. Crystal symmetry determination, i.e., Bravais lattice type, crystal system and/or space group symmetry from powder and single-crystal X-ray and electron diffraction data, has been studied.^7–16^ Furthermore, unit cell parameter determination from powder X-ray diffraction patterns has been addressed.^17,18^ *AlphaFold* serves as a tool for model building in protein crystallography.^19^ Regarding structure solution, a number of studies can be found. For example, DiffractGPT^20^ is able to solve crystal structures from powder X-ray diffraction data, but the results show that chemical information must be incorporated to enhance the prediction accuracy. In another study, the deep-learning framework XDXD^21^ was introduced for solving crystal structures from single-crystal X-ray diffraction data but using the molecular structure as input information. The PhAI neural network allows for ab initio phasing, meaning that no chemical information is needed as input. Another deep-learning work concerning ab initio phasing is related to structure solution from Patterson maps in simplified cases.^22,23^ Finally, there are opinions and work done in relation to connecting the classical and AI-based phasing methods.^24–26^

In this study, we present a neural network for ab initio phasing—GraPhAI. In contrast to our previous work,^1^ we have generalized phasing for all centrosymmetric crystal structures and for unit cells with a maximum cell dimension of 20 Å. We used the power of graph neural networks to represent the diffraction data more efficiently. The models were trained on balanced training sets of synthetic structures and evaluated using over 20,000 experimental crystal structures.

## Results and Discussion

### Representation of the Diffraction Data

Bragg diffraction data is a three dimensional (3D) discrete function with an integer domain and a real range representing diffracted intensities, i.e., structure factor amplitudes (*I* ∝ |*F*_obs_|^2^). Therefore, the data can be represented on a 3D tensor (Figure 1 left). Such data representation has several advantages: it is straightforward, the available list of Bragg reflections can be mapped onto the tensor very fast, and the tensor has a fixed shape for which the classical 3D convolution kernels can be applied. Indeed, the success of our previous work with PhAI^1^ has shown that diffraction data for deep learning can be represented in a 3D tensor form.

**Figure 1:**
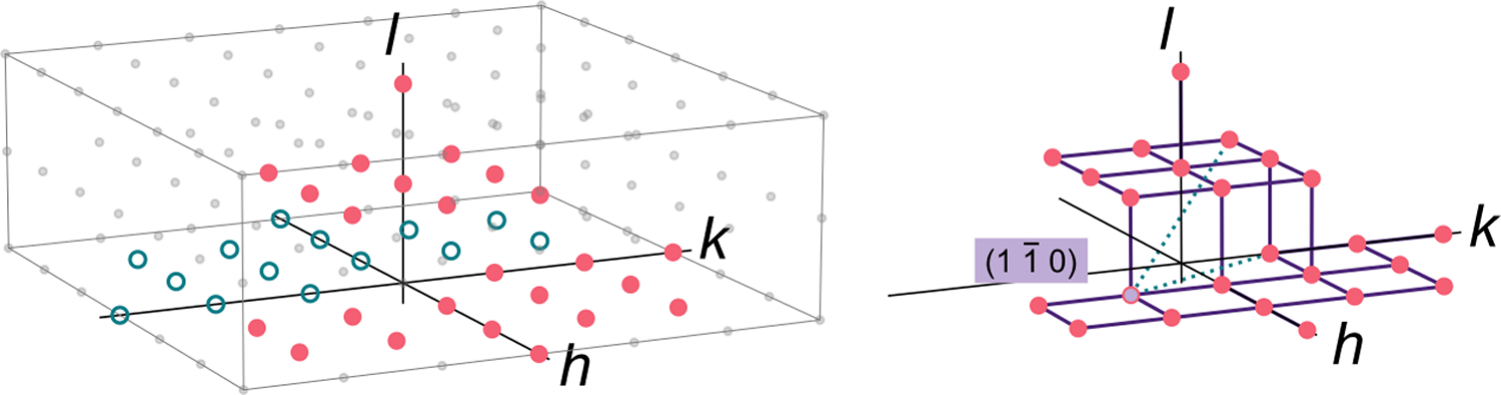
Diffraction data representation. Tensor (left) and graph (right) representations. The red dots represent a set of unique reflections (Laue class 1̅). The green dots represent reflections that are not symmetrically independent but can be represented on the given tensor. For the graph representation, all unique nodes are connected according to the lattice graph. For reflection (1 1̅ 0), additional connections are shown to complete the lattice graph for Laue class 1̅.

The available experimental diffraction data usually extend to a certain resolution limit d* effectively forming a sphere of reflections on the reciprocal lattice grid. Due to the symmetry of the diffraction pattern, even for the triclinic general case, only half of the reflections are unique. The unique reflections can be chosen so that *h* ∈ (−*∞*, *∞*), *k* ∈ (−*∞*, *∞*), and *l* ∈ (0, *∞*) with additional conditions for *hk*0 (*h* ≥ 0) and for 0*k*0 (*k* ≥ 0). For tensor representation with a maximal Miller index value of *n*, an array of shape (2*n* + 1, 2*n* + 1, *n* + 1) is required. A medium-size structure will have a few hundred to a few thousand reflections depending on the resolution. Meanwhile, the necessity to host reflections where any of the Miller indices can be around 20 is unavoidable. For *n* = 20, the tensor will have over 35,000 elements—most of which will have no structure factor information when representing data of average unit cell volume structures or low-resolution data. This motivates us to search for a more efficient diffraction data representation.

We propose to represent the diffraction data in a graph form. In this way, only the available Bragg reflections in each case can be used, avoiding the large fraction of absent data that is represented by zeros. Furthermore, data symmetry can be easily implemented. In Figure 1 (right), graph representation is shown. The graph nodes featuring structure factor information are connected to form a regular grid of the present reflections. This is effectively a lattice graph. Consequently, each node should have six neighbors, except for reflections neighboring (0 0 0) and those reaching the resolution limit. If a neighbor is missing due to the unique-only representation approach, the node can be connected to the symmetrically equivalent reflection. In Figure 1, one example is shown for the reflection (1 1̅ 0). It is connected with four other nodes within the set of the unique reflections. According to the lattice graph, reflection (1 1̅ 0) should also be connected with reflections (0 1̅ 0) and (1 1̅ 1̅), but both are absent in the chosen unique-only set. To correct for that, the missing connections can be made with the symmetrically equivalent reflections (0 1 0) and (1̅ 1 1), respectively. The reflection in question has therefore, all six neighbors for the information aggregation. Very importantly, this procedure, in fact, also ensures that all *m*-hop neighbors are necessarily connected and information aggregation can be performed at any depth. In terms of information aggregation, such a minimal graph is exactly equivalent to a twice as large lattice graph with all reflections included. Another interesting feature of representing the diffraction data in a graph form is the possible inclusion of additional connections between the nodes. For now, we have only studied purely lattice graphs.

Finally, a lattice graph is invariant with respect to a change of basis meaning that feature aggregation will produce numerically identical results irrespective of how the unit cell axes are labeled. This is an appealing thought, as it reduces the degrees of freedom in data representation.

### GraPhAI Neural Network Design and Training

Our proposed network – GraPhAI – is based on graph convolutions:

$$x_{v}^{\left(l+1\right)}=W_{1}^{\left(l\right)}x_{v}^{\left(l\right)}+\underset{u\in N\left(v\right)}{\sum }W_{2}^{\left(l\right)}x_{u}^{\left(l\right)}$$ 1

where $x_{v},x_{u}\in R^{N}$ are node vectors (input features or embeddings in hidden layers); N(v) is the neighborhood of node *v*; and $W_{1},W_{2}\in R^{N\times N}$ are learnable parameter matrices. Input features for each reflection **H** are the structure factor amplitude |*F*_**H**_| and the magnitude of the corresponding reciprocal lattice vector $\left|G_{H}\right|=d_{H}^{*}=\frac{1}{d_{H}}$ . The role of *d*_H_^*^ is to reintroduce spatial information into the graph representation, since graph convolutional networks do not take into account the order of neighboring nodes, unlike CNNs, which are inherently sensitive to spatial ordering.^27^ Furthermore, since lattice spacing *d*_**H**_ is dependent on unit cell parameters, this feature also contains information about the unit cell dimensions.

The graph convolutional network learns high-dimensional reflection embeddings; hence, it serves as an encoder. Similarly to the PhAI approach, we then project these embeddings to a fixed number of multilayer perceptron (MLP) tokens, which are passed to a decoder part consisting of MLP-Mixer layers,^28^ and finally, projected back to reflection space (Figure 2). The architecture is described in detail in the Methods section.

**Figure 2:**
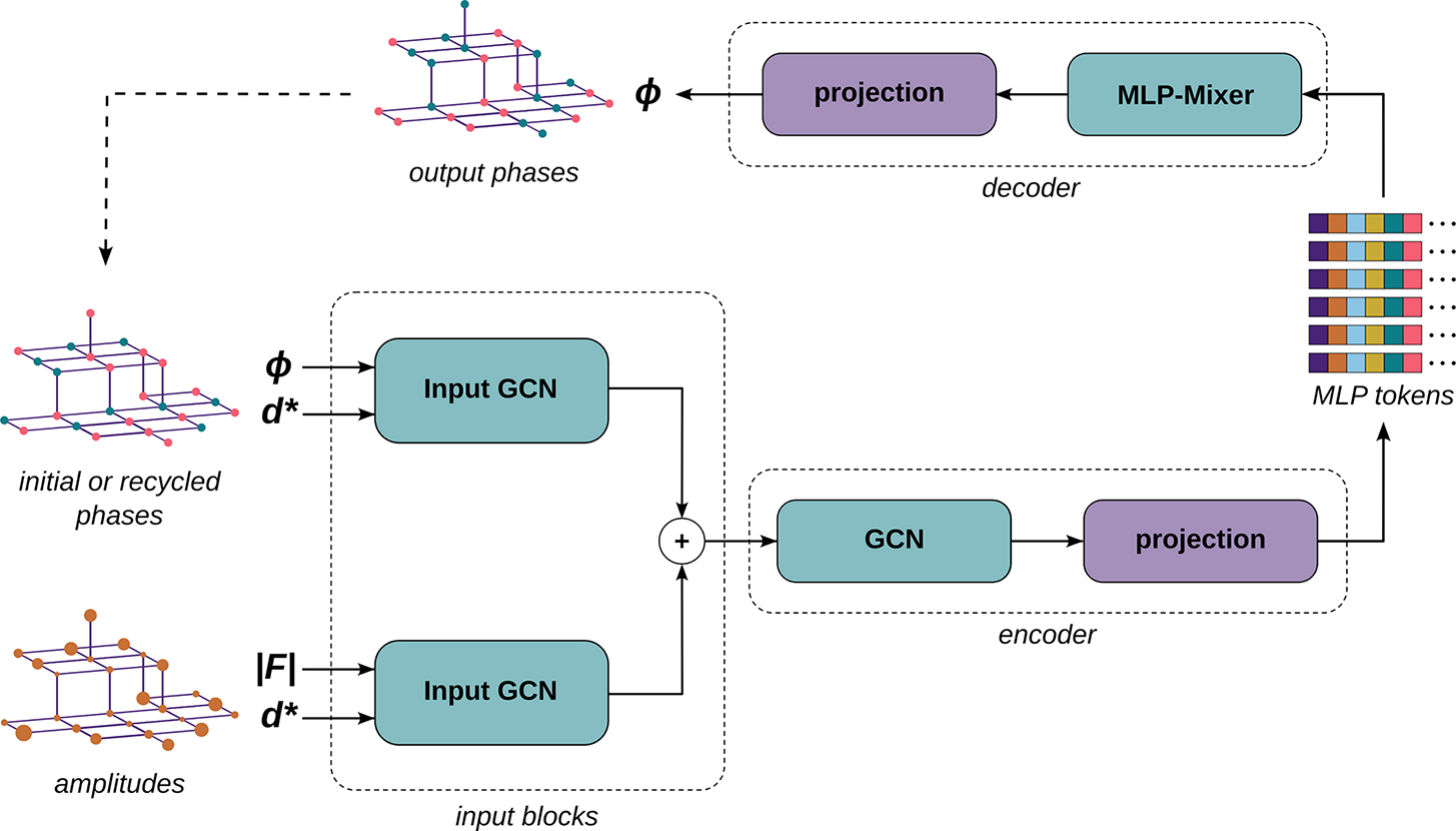
Diagram of GraPhAI neural network architecture. Amplitudes and initial (random) or recycled phases are treated with graph convolutional layers, which learn reflection embeddings. These are projected to MLP token space, passed through an MLP-Mixer, and projected back to reflection space to obtain the predicted phases.

The procedure of phase recycling, where the neural network is allowed to improve its predicted phases ϕ_**H**_ over several iterations starting from random values, is crucial for the performance of both PhAI and GraPhAI. We represent initial or predicted reflection phases with the same graph structure as described for the input amplitudes.

As far as training data are concerned, we adopted our previously reported training data generation strategy,^29^ extending it to the most general space group for centrosymmetric structures—*P*1̅. We trained the following GraPhAI models: 1) GraPhAI-10-v1 (the training data consisted of artificial *P*1̅ structures with the maximum unit cell dimension of 10 Å); 2) GraPhAI-20-v1-A (trained on *P*1̅ structures with the maximum unit cell dimension of 20 Å); 3) GraPhAI-20-v1-B (trained on *P*1̅ and *P*2_1_/*c* structures (60:40) with the maximum unit cell dimension of 20 Å).

### Performance of Model GraPhAI-10-v1

The performance of GraPhAI-10-v1 was tested by phasing diffraction data of ten thousand real crystal structures (see Figure S1 for more details on this data set). The Pearson’s correlation coefficient *r* = Corr­(ρ_predicted_, ρ_true_) between the phased and the true electron density map was used to assess the success. In Figure 3 (top), the fractions of solved crystal structures of model GraPhAI-10-v1 are depicted. We use 3 different thresholds to classify the structure as solved (*r* > 0.8, *r* > 0.9, and *r* > 0.99, respectively). Both good (*d*_min_ = 1 Å) and poor (*d*_min_ = 2 Å) resolution diffraction data were used for testing. All ten thousand structures were grouped by the compound class and by the heaviest element present. The results show that a vast majority of the phased density maps have an *r* > 0.9. Based on this criteria, 80–99% of the structures segregated by compound type can be solved from good resolution data. For low-resolution data, the respective success rate is also high. Furthermore, a large fraction of structures in each class shows *r* > 0.99, meaning that the corresponding solutions are very precise for data at *d*_min_ = 2 Å. The performance decreases for the inorganic structure subset, which we associate with the lack of purely inorganic artificial structures in the training data. In Figure 3 (top right), the same data are displayed segregated by the heaviest element found in the unit cell. Despite the success, this model has a limited practical relevance because it is intended for very small unit cell structures. The performance of our other GraPhAI models will therefore be explored in more detail.

**Figure 3:**
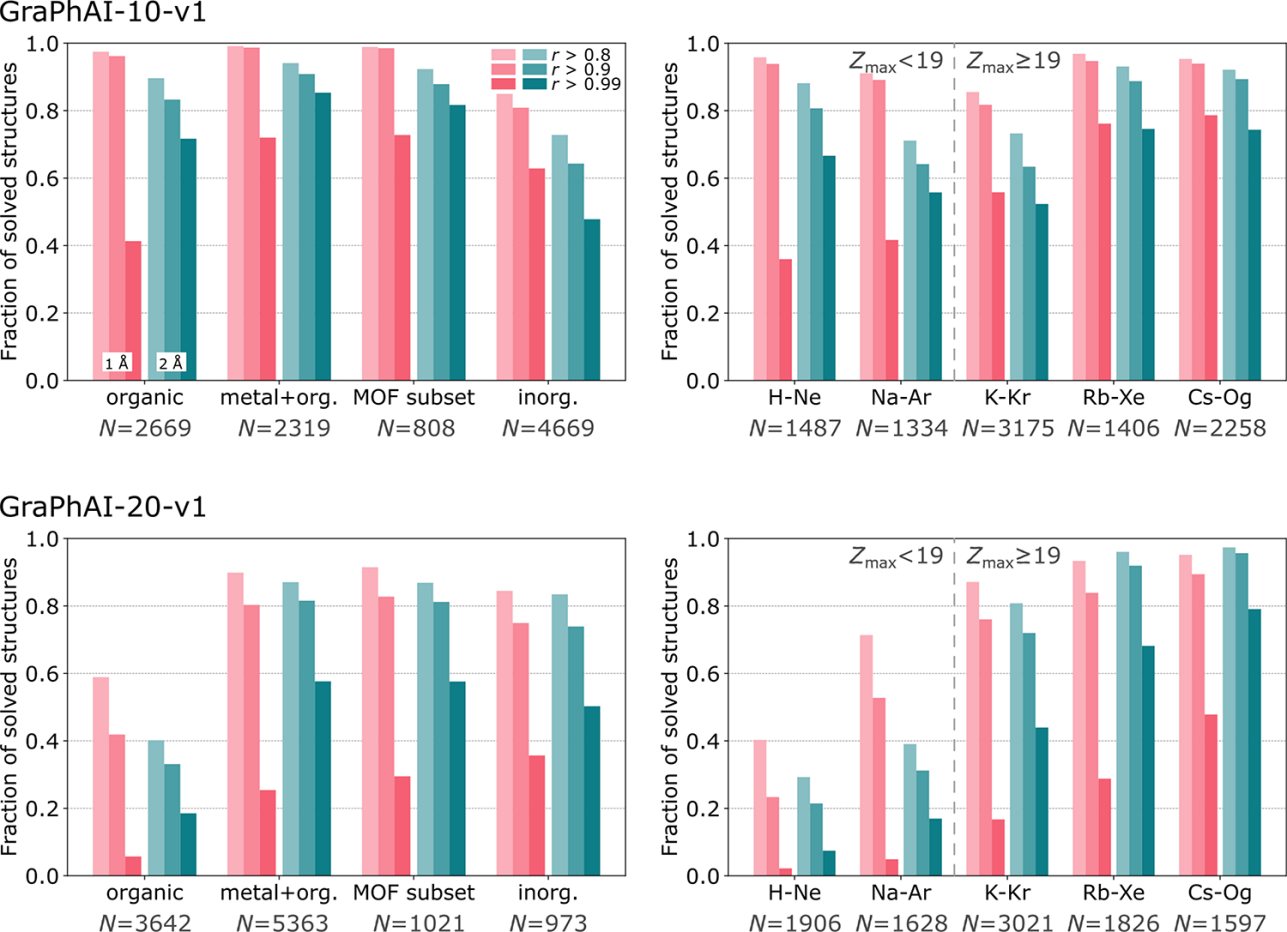
GraPhAI-10-v1 and GraPhAI-20-v1 performance. Each test set included approximately 10,000 centrosymmetric crystal structures with unit cell dimensions up to 10 or 20 Å. Left: Structures partitioned by compound class. Right: Structures partitioned by heaviest element present. The red bars correspond to 1 Å resolution data; the green bars correspond to 2 Å resolution data. Darker shade corresponds to a stricter criterion for assuming the structures to be solved.

### Performance of Models GraPhAI-20-v1-A and GraPhAI-20-v1-B

Models GraPhAI-20-v1-A and GraPhAI-20-v1-B differ by the training domains used. In Figure 3 (bottom), combined results of phasing a ten thousand real structure data set with the largest unit cell dimension of 20 Å are presented (see Figure S1 for more details on this data set).

Most structures containing metal atoms and organic molecules (coordination compounds, organometallic compounds, and MOFs) as well as inorganic structures can be solved with GraPhAI-20-v1. The map accuracy is generally high, especially when phasing low-resolution data. In Figure 4, selected examples of the reconstructed electron density of the molecules/coordination complexes are shown. Note that for structures containing particularly heavy atoms, their density maps can be rather noisy even when the predicted phases match with the true phases perfectly. This is due to the Fourier termination effects.

**Figure 4:**
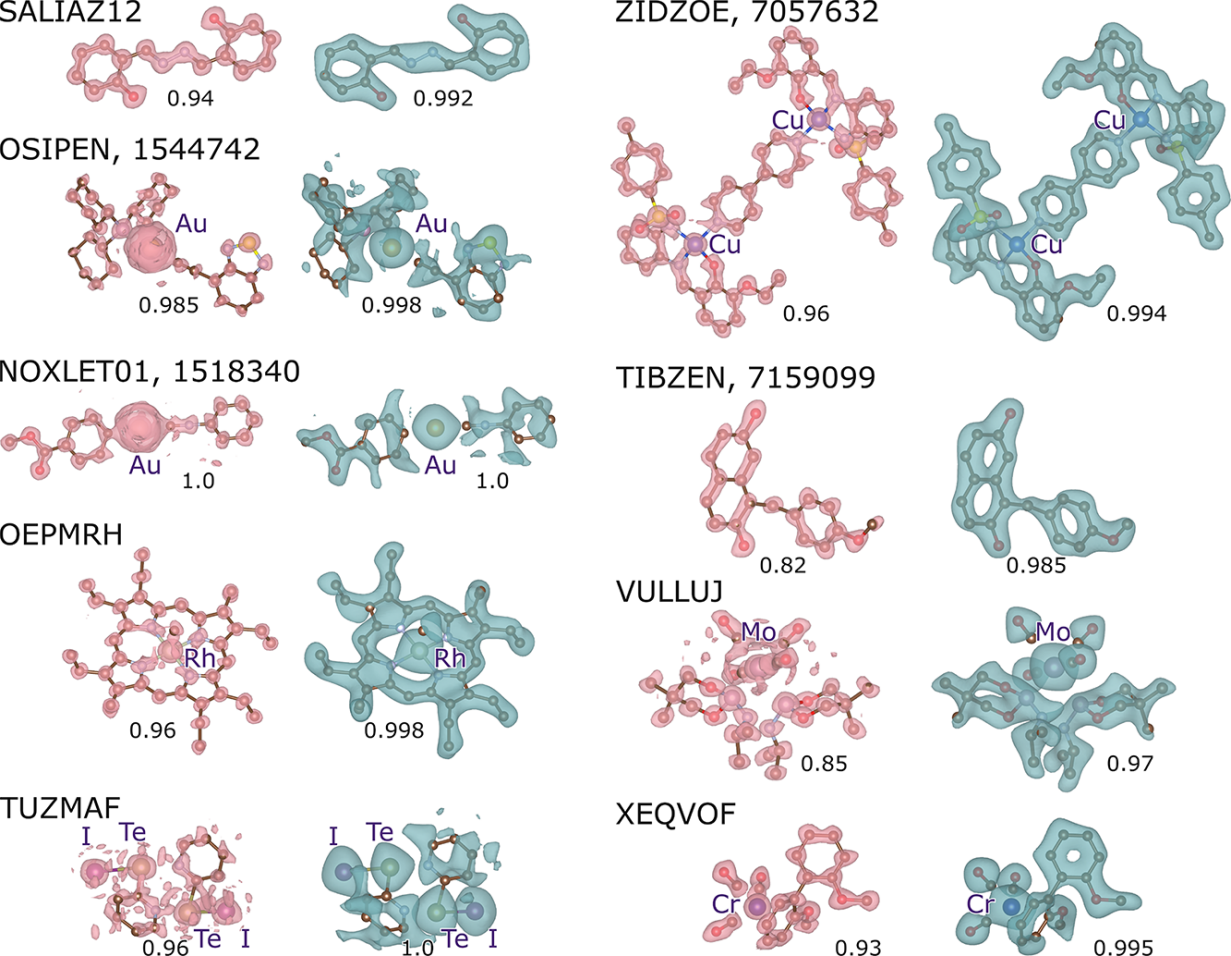
Selected examples of the reconstructed electron density maps. Red: 1 Å resolution, green: 2 Å resolution. For each structure, their CSD and COD identifiers are given along with the correlation coefficient *r* values between the predicted and true electron density maps. Particularly heavy atoms in the structure are indicated with atomic symbols.

To highlight the breakthrough in phasing low-resolution diffraction data, we have performed some benchmarking in which the same structure set is phased with charge flipping^30^ and standard direct methods.^31^ The results are presented in Figure S2. For charge flipping and standard direct methods, the performance is virtually the same in each subset regardless of how the test structures are grouped. For GraPhAI-20-v1 models, there are more pronounced differences in phasing performance based on the differently segregated data. This is further discussed in more detail below.

### The Effect of Atomic Composition

Models A and B tackle the phasing of purely organic structures poorly, particularly if the organic molecules contain only light elements. This was not the case for small-unit-cell centrosymmetric structures phased with GraPhAI-10-v1. We see that generalizing the phase problem solution for light-element structures does not scale well in the unit cell volume dimension with the present architecture/hyperparameters used.

The major difference in diffraction data for light-element structures (or equal element structures) and structures containing atoms with different scattering powers is the intensity distribution, as well as the phase relationships. For the former, the structure factor amplitudes are typically very large for a few low-angle reflections, whereas for the latter, the amplitudes are more evenly distributed. Furthermore, phases behave rather randomly in equal-atom structures, whereas they are dominated by the heavy atom positions in heavy atom structures.^32,33^

### The Effect of Space Group Symmetry

One of the objectives of this study was to try to generalize the solution of the phase problem for all centrosymmetric structures. We discovered that generalization to other symmetries is good if only *P*1̅ structures are in the training domain (model GraPhAI-20-v1-A, see Figure 5). However, better results can be obtained when *P*2_1_/*c* structures are included in the training data (model GraPhAI-20-v1-B) due to their high frequency among the crystal structures. Well over 50% of the centrosymmetric structures crystallize in *P*2_1_/*c* or its supergroups, e.g., *C*2/*c* or Pbca.^34^ In Figure S3, the graphs of *r*_A_ vs *r*_B_ are given. It shows that model B deals better with structures in *P*2_1_/*c* and its most common supergroups *C*2/*c* and Pbca (Figure S3 middle). Contrary, model A can successfully phase a fraction of structures in *P*1̅ for which model B fails. No particular tendencies are observed for structures in space groups other than *P*1̅ or *P*2_1_/*c* (Figure S3 right), i.e., none of the models shows increased success in phasing this respective structure subset. Nevertheless, for model A, the maps are more accurate.

**Figure 5:**
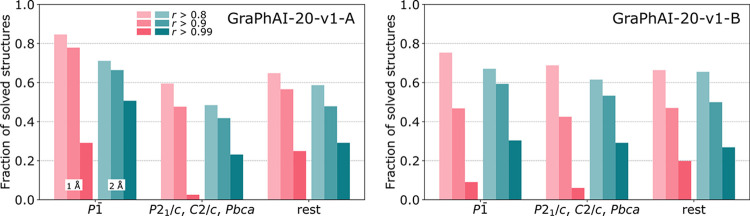
Generalization outside the space group domain used for training data. Results of phasing the test set of approximately 10,000 centrosymmetric crystal structures with unit cell size up to 20 Å partitioned by the space group symmetry. Left: Model GraPhAI-20-v1-A (trained on *P*1̅ structures only). Right: GraPhAI-20-v1-B (trained on *P*1̅ and *P*2_1_/*c* structures). The red bars correspond to 1 Å resolution data; the green bars correspond to 2 Å resolution data. Darker shade corresponds to a stricter criterion for assuming the structures to be solved.

### The Effect of Unit Cell Volume

In Figure 6 top, the results are segregated by the unit cell volume showing violin plots of *r* values for 500 Å^3^ volume bins. The histogram of the unit cell volume distribution of the test set is given along with the fitted log-normal probability density function, as found for all centrosymmetric structures in the structure databases (the latter function was also used for the training set generation^29^). For phasing 1 Å data, the distributions of *r* values become broader with increased unit cell volume, meaning that the phased density maps become progressively less accurate. A similar trend is observed in Figure 6 (bottom left), where the fraction of solved crystal structures (*r* > 0.8) are given in a similar plot. Nevertheless, a typical structure of up to around 2500 Å^3^ unit cell volume can mostly be solved with a high accuracy.

**Figure 6:**
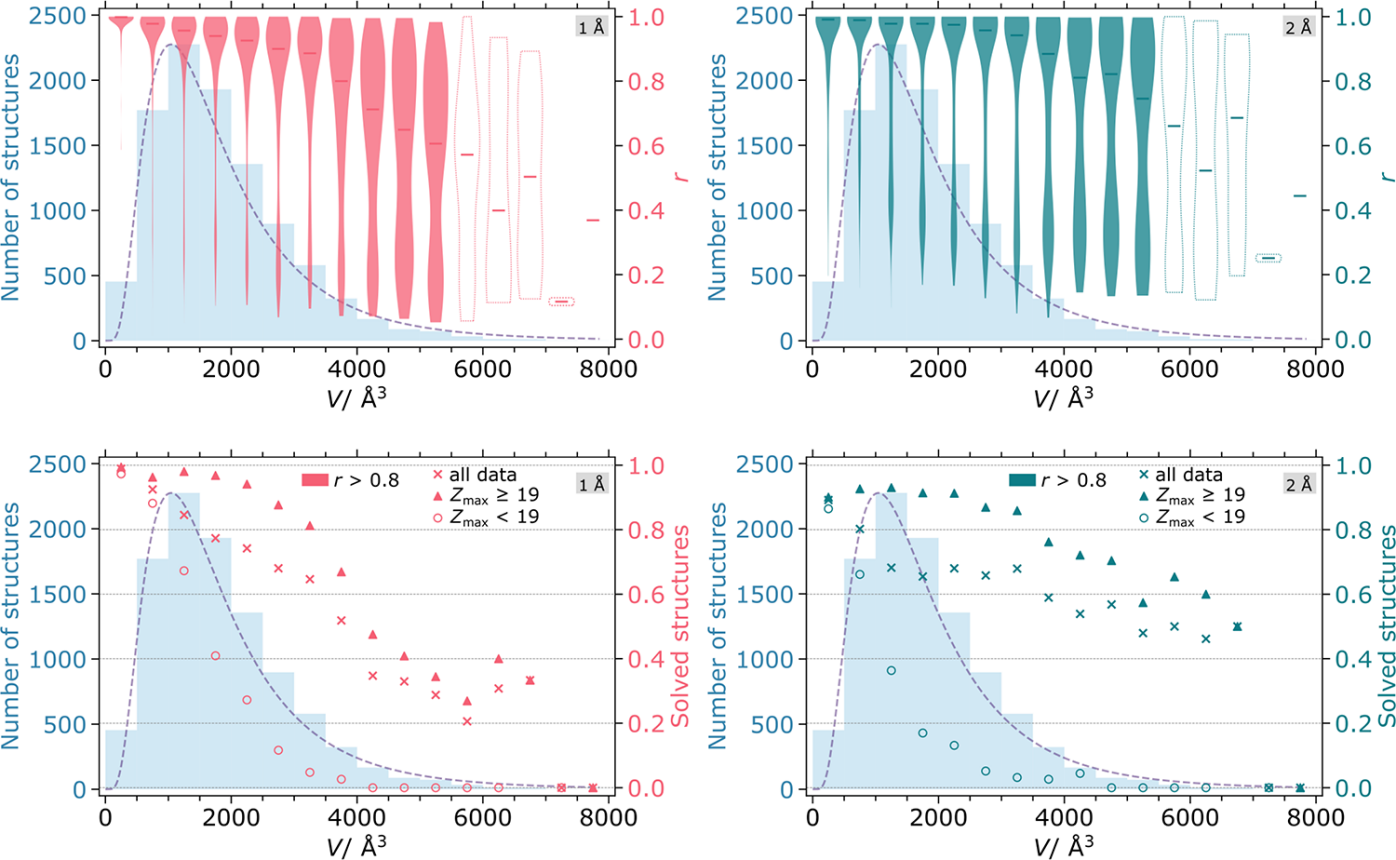
Phasing results of GraPhAI-20-v1 partitioned by the unit cell volume (*N* = 10 000). Left: 1 Å data, right: 2 Å data. Top violin plots in each volume bin show the distribution of *r* values with the medians given. The data are calculated from a small number of structures at higher volume ranges. Markers in the bottom graphs show the fraction of solved structures (*r* > 0.8) in each volume bin for all structures, structures with *Z*_max_ ≥ 19, and structures with *Z*_max_ < 19. The dashed line shows the distribution of the unit cell volume in the training set.

Somewhat different trends are observed when phasing 2 Å resolution data. The map accuracy remains largely high also at higher unit cell volumes and starts to fail only at around 4000 Å^3^ volume (Figure 6 top right). The fraction of solved structures has a less pronounced volume dependence (Figure 6 bottom right). For both tested resolutions, once again, the significant difference between phasing structures with light atoms (*Z*_max_ < 19) and heavy-atom structures (*Z*_max_ ≥ 19) emerges. The data in Figure 6 (bottom right) show that a typical heavy-atom structure with a unit cell volume < 2500 Å^3^ can be solved from low-resolution data in over 90% of cases. This is relevant for solving, for example, MOF structures.

### The Effect of Permuting Unit Cell Axis

We noted earlier that representing diffraction data in the graph form reduces the degrees of freedom, since information aggregation is invariant with respect to unit cell axis permutations. However, GraPhAI-v1 models are not yet fully invariant in this respect, as the projection step from the reflection space to the MLP token space and back (Figure 2) also implicitly encodes the spatial structure of the diffraction data. Therefore, there is no reason not to consider all possible permutations of the unit-cell axis during neural network inference.

Including the identity, there are 12 variants of an arbitrary unit cell with permuted unit cell axes while keeping the coordinate system right-handed and not changing the Euclidean norms of the given unit cell vectors. For higher-symmetry space groups, not all 12 variants are unique. Initially, we phased the testing set of ten thousand structures without permuting the unit cell axis. Then, structures with *r* < 0.8 were phased again considering all 12 unit cell variants with permuted axis. The effect was not major, but a substantial number of additional data sets could be successfully phased (Figure S4). All results reported before take into account axis permutations.

### The Effect of Data Representation

In order to quantify the influence of diffraction data representation on the computational cost of training and on model accuracy, we also trained PhAI-20, a CNN model with an architecture identical to that of PhAI except for expanding the tensor size to (41, 41, 21). The training data set was the same as that for model GraPhAI-20-v1-A, and the two models were trained on identical hardware (AMD Instinct MI250X GPUs; see the Methods section). This allows for a direct comparison of training times.

The full training workflow (150 million structures) took 3344 GPU hours for model PhAI-20 and only 1840 GPU hours for model GraPhAI-20-v1-A. Furthermore, the GraPhAI-20-v1-A model performs slightly better than PhAI-20 on inference with the same test set (Figure S5). We must explicitly state that these results should not be taken as a broad comparison of GNN and CNN architectures since in both cases further hyperparameter optimization may be possible. Rather, by isolating the variable of diffraction data representation, we demonstrate that the graph form is at least as expressive as the tensor form while also decreasing training time by a factor of 1.8 for this particular training data domain.

### X-ray Diffraction Data

The GraPhAI models were trained on synthetic crystal structures, specifically on calculated diffraction data based on these structure models. We already showed that phasing real structures based on calculated diffraction data is successful. Now, we will assess the *observation gap*. The calculated training diffraction data are affected by modeling simplifications, like the use of independent atom approximation, simplified atomic displacement description, excluded additional physical effects (e.g., resonant scattering), and the absence of systematic and random errors. For these reasons, it is important to also evaluate GraPhAI model performance on experimental diffraction data.

A total of 937 data sets with experimentally measured amplitudes (|*F*_obs_|) for structures within the current GraPhAI-20-v1 domain were found (see Figure S1 for more details on this data set). For reference, we also tested this set of 937 structures with calculated diffraction data. Around 200 structures could not be solved either from the calculated diffraction data or from the experimental diffraction data. We analyzed the 731 cases in which the solution could be obtained from the calculated data. Over 90% of those could also be solved from the experimental diffraction data. To understand better why around 10% of the 731 structures could not be solved from the experimental data, we first looked at the correlation coefficient between |*F*_obs_| and |*F*_calc_|. In Figure 7, (left), a histogram of Corr­(|*F*_obs_|, |*F*_calc_|) is given. The correlation is mostly very good, meaning that the reported structure models correspond to the experimental data very well. There is a significant portion of data where Corr­(|*F*_obs_|, |*F*_calc_|) is slightly lower than the mode, and these structures can be solved by GraPhAI. There appears to be no difference between calculated diffraction data (using a simplified crystal structure model and diffraction physics) and experimental data (with random and systematic errors included), as far as the operation of GraPhAI is concerned. In fact, correct phases can be obtained even from data with Corr­(|*F*_obs_|, |*F*_calc_|) < 0.7, as illustrated by the inset plots in Figure 7 (left).

**Figure 7:**
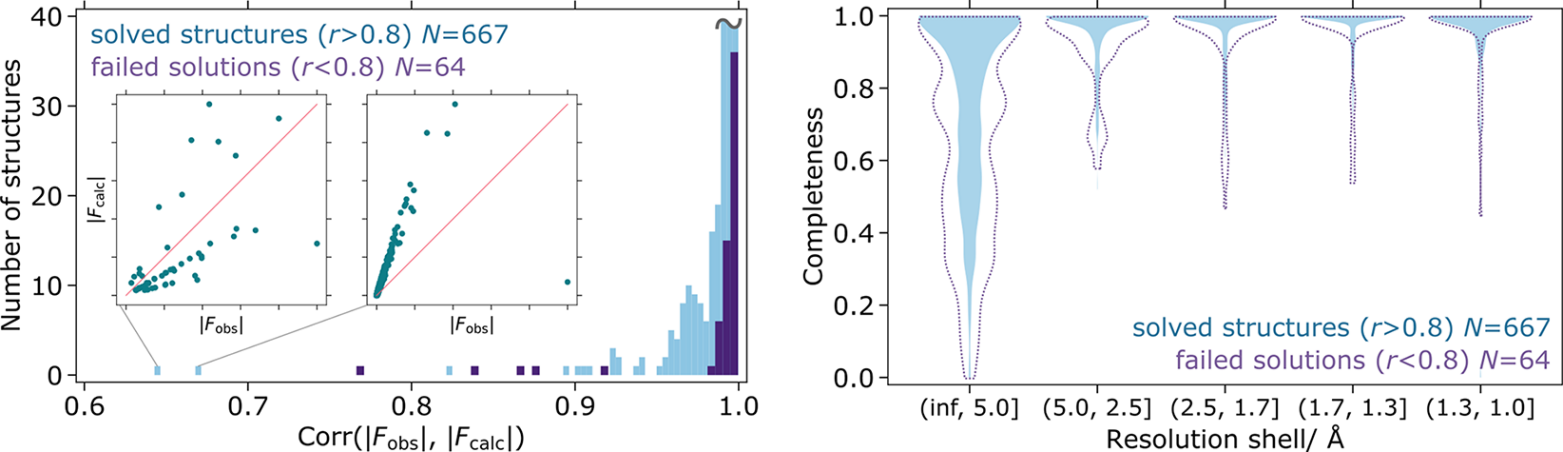
Phasing of experimental X-ray diffraction data. Left: Histograms of Corr­(|*F*_obs_|, |*F*_calc_|) for solved structures and failed solutions with GraPhAI-20-v1 from experimental X-ray diffraction data (*N* = 731). Right: Violin plots of the experimental data completeness in resolution shells.

We suspected that the slightly lower performance on the experimental data could be attributed to the data completeness. In Figure 7 (right), violin plots of the data completeness across the resolution shells are given. The completeness, especially in the lower shells (higher *d*), is pronouncedly lower for the experimental data sets that failed to be phased with GraPhAI. Apparently, a critical amount of missing reflection amplitudes in the experimental data (but present in the calculated data) has hindered GraPhAI from obtaining the solution in those 64 cases. Meanwhile, there are cases present with imperfect data completeness also in the set of 667 successfully phased experimental data.

### Electron Diffraction Data

We also tested 102 electron diffraction data sets. While the phased structures are within the domain of the present GraPhAI models, the experimental diffraction data these structures are represented by differ. First, the atomic form factors differ. GraPhAI was trained using X-ray atomic form factors for calculating the diffraction data. Second, electron diffraction data are heavily influenced by dynamical effects, meaning that the correlation between the observed structure factor amplitudes (|*F*_obs_|) and those calculated from a correct structure model using the kinematical theory of diffraction (|*F*_calc_^e–^|) is not as good as in the case of X-ray diffraction (Figure 8, top left). Third, the data are often low completeness (Figure 8, top right). For these reasons, a comparable performance as for the X-ray diffraction data is not to be expected at this point. Phasing of most of the electron diffraction data sets indeed failed, but we demonstrate two examples where it was successful.

**Figure 8:**
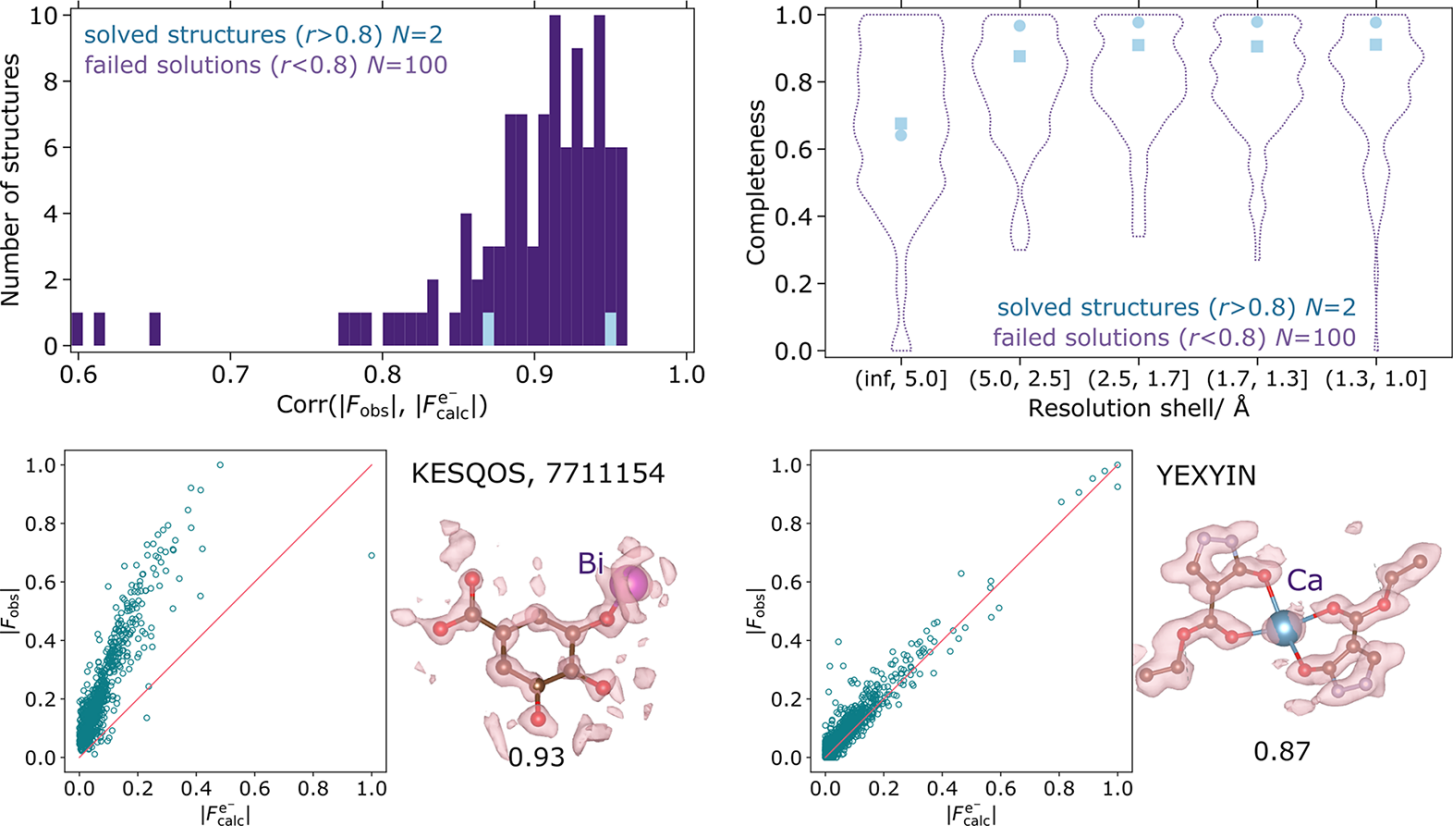
Phasing of experimental electron diffraction data (*N* = 102). Top left: Histograms of Corr­(|*F*_obs_|, |*F*_calc_^e^–^^|) for solved structures and failed solutions with GraPhAI-20-v1 from experimental electron diffraction data. Top right: Violin plots of the experimental data completeness in resolution shells. Bottom: Two successfully phased electron diffraction data sets showing average data plots (|*F*_calc_^e^–^^| vs |*F*_obs_|), as well as the resulting electrostatic potential maps of the chemical entities and the respective *r* values between the true and the phased maps.

In Figure 8 (bottom), the electron diffraction data correspondence to the structure models after data averaging is given together with the electrostatic potential maps obtained from the GraPhAI-20-v1-B output phases. The correlation coefficients Corr­(|*F*_obs_|, |*F*_calc_^e^–^^|) are substantially lower than those in the case of X-ray diffraction data (Figure 7, left), but the completeness is high compared to most of the other tested electron diffraction data sets (Figure 8, top right). These observations suggest that data completeness is a more crucial problem for the developed neural networks than the input structure factor amplitude correspondence to the kinematical diffraction data of some structure model.

## Conclusions

We have presented a graph neural network, GraPhAI, for the solution of the crystallographic phase problem, which is based on a graph representation of the diffraction data. This form of representation considerably saves the compute. The trained GraPhAI-v1 models were capable of ab initio phasing of centrosymmetric structures with a maximum unit cell dimension of 20 Å. The solutions were correct for a large fraction of tested the 10,000 real crystal structures, particularly when a low-resolution (2 Å) data were used. The success was above 80% for structures containing an element with *Z* ≥ 19. A lower performance was observed for light-element structures. We noticed that models trained on structures in *P*1̅ are capable of solving centrosymmetric structures in other space groups, but the performance for solving structures in *P*2_1_/*c* and its supergroups can be somewhat improved when structures in this space group are included in the training set. We also tested all available experimental diffraction data and did not detect a significant observation gap. Therefore, the presented GraPhAI models have practical relevance for phasing typical so-called small-molecule crystal structures from low-resolution data.

## Methods

### Training Structure Generation

Crystal structures for training were generated by the approach described previously.^29^ The unit cell volume was sampled from a log-normal distribution fitted to values from a uniquely merged Crystallography Open Database (COD)^35^ and the Cambridge Structural Database (CSD)^36^ for structures in the cell parameter ranges 4 Å ≤ *a*_*n*_ ≤ 10 Å and 4 Å ≤ *a*_*n*_ ≤ 20 Å. The fitted parameters were μ = 6.04, σ^2^ = 0.394 for the former, and μ = 7.35, σ^2^ = 0.633 for the latter (with unit cell volume expressed in Å^3^). After sampling the volume, lattice basis vectors were calculated to obtain either a triclinic unit cell for *P*1̅ structures or a monoclinic unit cell for *P*2_1_/*c* structures.

Unit cell contents were generated using the artificial molecule method. Element distributions for atoms in general and special positions, as well as bounds for *U*_iso_, the volume per atom, bond lengths, and the hydrogen atom fraction were taken from the previous work. The probability of finding an atom in a special position was chosen as 0.015 for *P*1̅ and 0.05 for *P*2_1_/*c*.

### Diffraction Data Generation

For each training structure, diffraction data were calculated on-the fly. Structures in *P*2_1_/*c* were transformed to *P*1. A resolution was sampled uniformly between 0.25 Å^–1^ and 0.5 Å^–1^ (1/2*d* = sinθ/λ) and then a list of unique *h*, *k*, *l* indices was generated according to Laue class 1̅. Atomic form factors were calculated as sums of Gaussian terms using the constants given in the International Tables for Crystallography.^37^ Structure factors were then calculated on a 3D tensor along the dimensions of the given Miller indices, space group symmetry elements, and atomic positions of the asymmetric unit. Summing over the latter two dimensions yielded a list of the needed structure factors. The procedures were adapted to run the calculations on the GPU. In addition to |*F*_calc_|, d* values for each reflection were obtained using the reciprocal metric tensor. Precalculated maps were used to generate the lattice graphs of the diffraction data. Before passing to the neural network for training or inference, both input features were rescaled to the interval [0, 1] at the sample level, i.e., divided by the respective maximum value for each structure.

### Neural Network Training

The encoder part of GraPhAI architecture (Figure 2) consists of graph convolutional blocks, which are composed of a graph convolution layer (GraphConv, eq 1), layer normalization, and GELU activation. Residual (skip-sum) connections are used, skipping two GCN blocks (Figure S6). The decoder part consists of MLP-Mixer blocks whose architecture was kept the same as that in the PhAI neural network.^1^ Architectural hyperparameters are summarized in Table S1.

All models were trained on generated structures (150 million for models GraPhAI-10-v1 and GraPhAI-20-v1-A, 180 million for model GraPhAI-20-v1-B). For model GraPhAI-10-v1, the structures were generated on-the-fly during training, while for models GraphAI-20-v1-A and GraPhAI-20-v1-B, the structures were generated prior to training and stored on disk. Calculation of structure factors and generation of the reflection graph were done at the time of training in all cases. Phase recycling was done for three cycles, starting from random phase values and calculating parameter gradients only on the last cycle. Training was done on NVIDIA GeForce RTX 5090, NVIDIA GeForce RTX 4090, NVIDIA A10G, or AMD Instinct MI250X GPUs, minimizing a weighted sum of binary cross-entropy loss using the AdamW optimizer with a batch size of 512, weight decay 1 × 10^–6^, and initial learning rate 4 × 10^–3^. Learning rate was reduced manually on loss plateau, first to 5 × 10^–4^ and then to 5 × 10^–5^. A validation set was also used, consisting of 150,000 structures generated by the same approach as training structures. No divergence of validation and training loss was observed at any point (Figure S7).

### Real Crystal Structure Processing

Crystal structures for model testing were taken from the uniquely merged COD and CSD databases in the cell parameter ranges 4 Å ≤ *a*_*n*_ ≤ 10 Å and 4 Å ≤ *a*_*n*_ ≤ 20 Å (*N* = 10,000 each, sampled randomly from all centrosymmetric structures in the given ranges). To ensure alignment with the training data, structures in space group 14 in nonstandard settings (*P*2_1_/*n* and others) were transformed to *P*2_1_/*c* for model GraPhAI-20-v1-B. Furthermore, *P*2_1_/*c* supergroup structures were transformed so that the positions of the common symmetry elements with respect to the unit cell origin aligned with *P*2_1_/*c*. All structures were afterward transformed to *P*1.

Simulated diffraction data were calculated at 1.0 and 2.0 Å resolution for each structure, transformed to a graph form as described previously, and passed for phasing. The associated structure model was used to assess the phasing success.

### Experimental Diffraction Data Processing

For all structures within the relevant domain, their Fourier coefficients files (.fcf) were searched in the COD. In 937 cases, the lists of experimental ‘F_squared’ information were present. Even though the.fcf should mostly contain merged data, all files were processed through our own data merging routines. All given reflections were reindexed according to the respective Laue class and the unique wedge of the reciprocal space chosen according to our conventions. The equivalent |*F*_obs_|^2^ (if present) were then averaged. Since formally data of *P*1̅ symmetry are necessary, the symmetrization was performed according to the space group symmetry. The associated lattice parameters were then used to generate a list of *h*, *k*, *l* indices up to a 1 Å resolution. Finally, the corresponding amplitude tensor with zero values was initialized and the experimental data (in the form of $\sqrt{|F_{\mathrm{obs}}{|}^{2}}/\max\left(\sqrt{|F_{\mathrm{obs}}{|}^{2}}\right)$ or 0 if |*F*_obs_|^2^ < 0) were mapped onto this tensor, i.e., experimental data with *d* < 1 Å were discarded and in case some reflections were not present in the data (not measured or systematically absent reflections), those were retaining zero values. The data were transformed into a graph form including also d* values for each reflection and passed for phasing. The associated structure model was used to assess the phasing success. The electron diffraction data sets were downloaded from the CSD and processed similarly.

### Neural Network Inference

On inference, the neural network outputs were treated with a sigmoid function, rounded, and multiplied by π to obtain a phase of 0 or π rad. The correlation coefficient between the phased and true electron density maps was calculated as follows:

$$r=\max_{t\in T}\;\frac{\sum_{H}|F_{H}{|}^{2}\cos(\phi_{H}-2\pi H\cdot t-{\hat{\phi }}_{H})}{\sum_{H}|F_{H}{|}^{2}}$$ 2

where |*F*_**H**_| is either $\sqrt{|F_{\mathrm{obs},H}{|}^{2}}$ if phasing experimental data or the reflection amplitude calculated from the structure model; ϕ_**H**_ is the true phase calculated from the structure model; ϕ̂_**H**_ is the predicted phase; **t** is a translation vector describing an alternate choice of origin in real space; and *T* is the set of all possible origins, i.e., all unique inversion centers in the given space group.

Phase recycling on inference was done for 10 cycles, calculating *r* for the predicted phases after each cycle, starting once with all zero initial phases and four times with different random initial phases. The procedure was repeated for each of the 12 unit cell axis permutations (see Results and Discussion). The final *r* value reported for each structure is the maximum from all runs. We consider this to be an accurate metric of model performance, since in practice successful and unsuccessful phasing runs can be easily distinguished even for an unknown structure in the course of electron density map interpretation and subsequent crystal structure refinement. The phased electron density map visualization was done with the program VESTA.^38^

## Supplementary Material



## Data Availability

GraPhAI-v1 code (including training data) under the MIT license (10.5281/zenodo.18835283), GraPhAI-v1 code (excluding training data) under the MIT license (https://github.com/Project-Lonsdale/GraPhAI-v1/).

## Notes

GraPhAI_SI.pdf: statistical description of the training and experimental data, additional figures summarizing the results of phasing the test structures, additional details of the neural network architecture and validation loss (PDF)
