# Supplementary material for: GraPhAI: Neural Networks for Solving Centrosymmetric Crystal Structures
Source: J Am Chem Soc. 2026 Jun 30;148(27):28754–63. doi: 10.1021/jacs.6c05607 (PMC13383728; doi:10.1021/jacs.6c05607)
Supplement: Supplementary file 1 [file ja6c05607_si_001.pdf]

SUPPORTING INFORMATION

GraPhAI: Neural Networks for Solving Centrosymmetric Crystal Structures

Džonatans Miks Melgalvis[1] and Toms Rekis[1,2*]

[1*]Faculty of Medicine and Life Sciences, University of Latvia, Jelgavas iela 1, Riga, LV1004, Latvia.

[2]Institute for Inorganic and Analytical Chemistry, Goethe-University Frankfurt, Max-von-Laue Straße 7, Frankfurt am Main, 60438, Germany.

*Corresponding author(s). E-mail(s): toms.rekis@lu.lv;
Contributing authors: dzonatans.melgalvis@lu.lv;

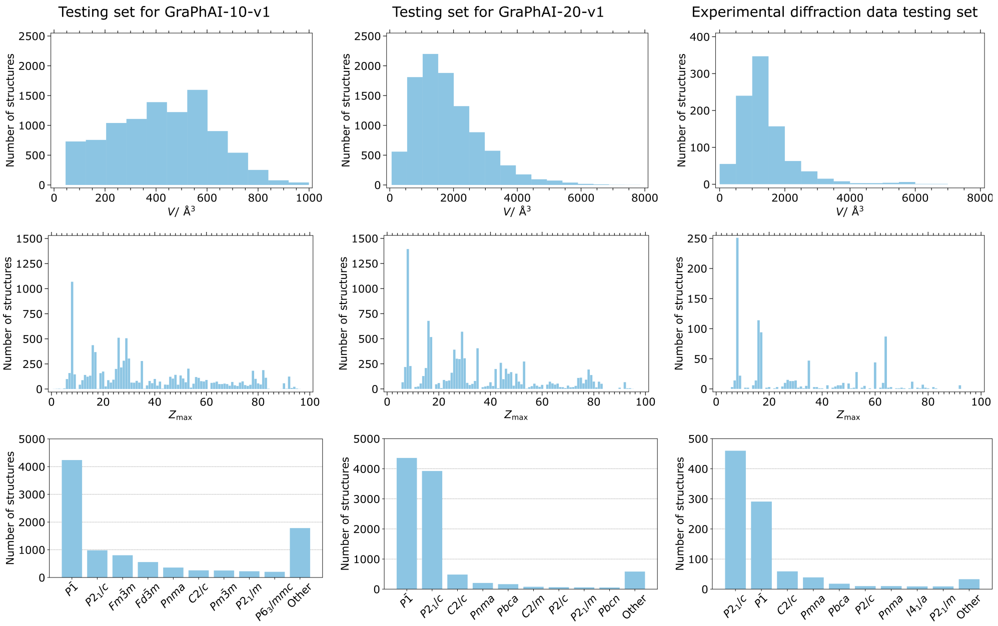

Fig. S1 Unit cell volume, Z_{max}, and space group frequencies of the test sets. First two each consists of approx. 10 000 real centrosymmetric crystal structures randomly selected from the uniquely merged Crystallography Open Database and the Cambridge Structural Database based on the maximal unit cell length of 10 or 20 Å. The experimental X-ray diffraction data set consists of 937 examples.

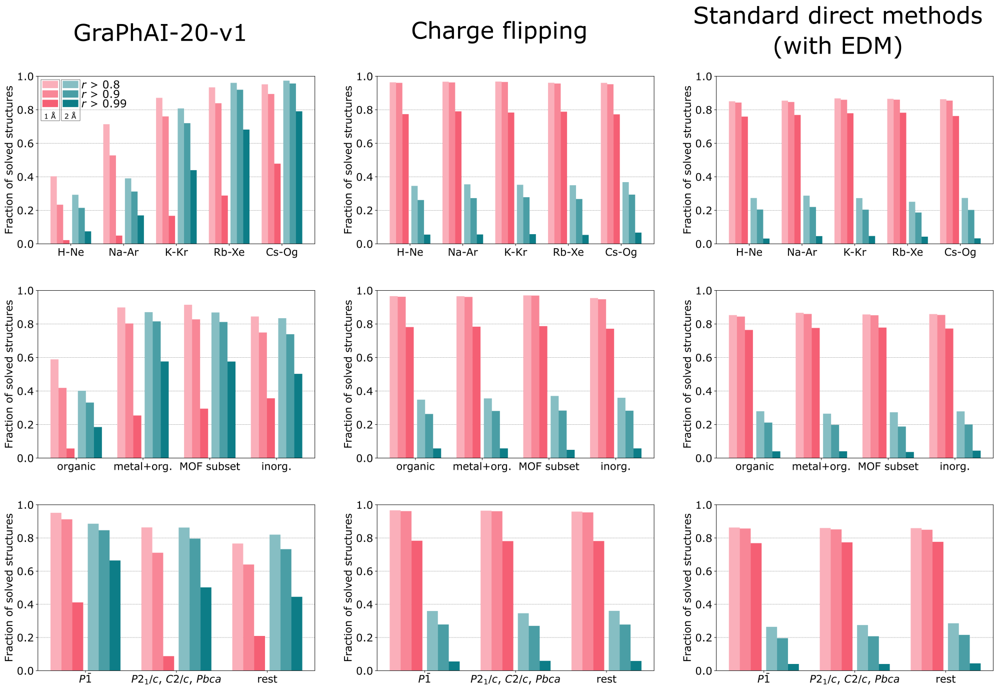

Fig. S2 Comparison of normal (red bars) and low-resolution (green bars) diffraction data phasing with different methods: 1) GraPhAI-20-v1 (versions A and B combined); 2) charge-flipping (program Superflip); 3) standard direct methods (program Sir2021). The test set consists of 10 000 centrosymmetric crystal structures with unit cell size up to 20 Å. In each row, the data set is segregated by different criteria.

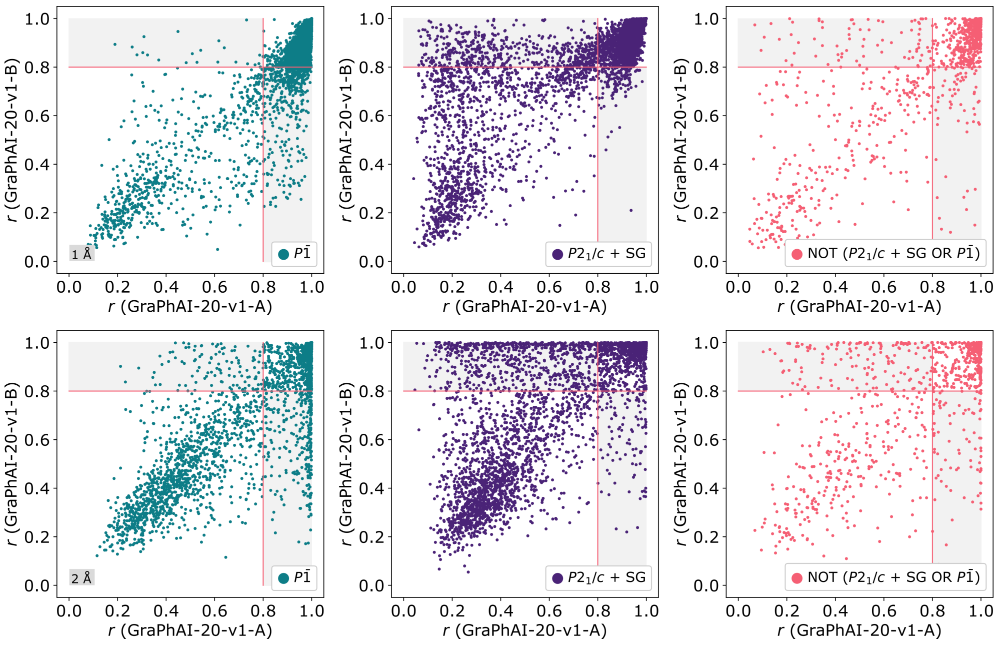

Fig. S3 Comparison of the performance of models GraPhAI-20-v1-A and GraPhAI-20-v1-B. The 10 000 testing set is segrageted by space group: $P\bar{1}$ (left); $P2_1/c$ and its most common supergroups (center); the rest of the space groups (right). Results of phasing the structures from 1 (top row) and 2 Å (bottom row) diffraction data are shown. The shaded areas correspond to cases where one of the models fails but the other one succeeds based on criterion $r > 0.8$.

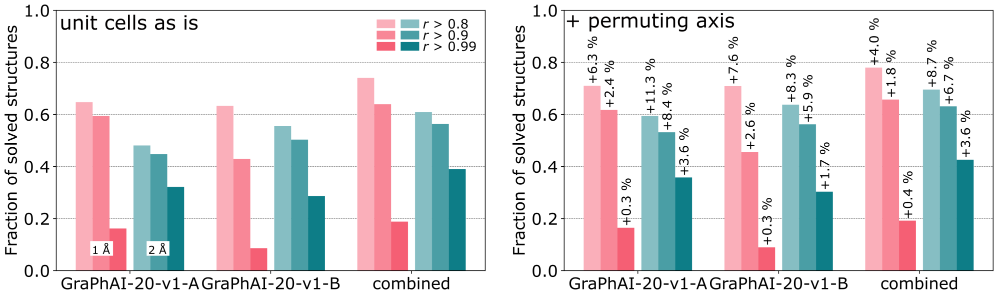

Fig. S4 Results of phasing the test set of 10 000 centrosymmetric crystal structures with unit cell size up to 20 Å. Left: initial results phasing the data with unit cells as given. Right: results after phasing previously failed cases ($r < 0.8$) again including 12 axis permutations.

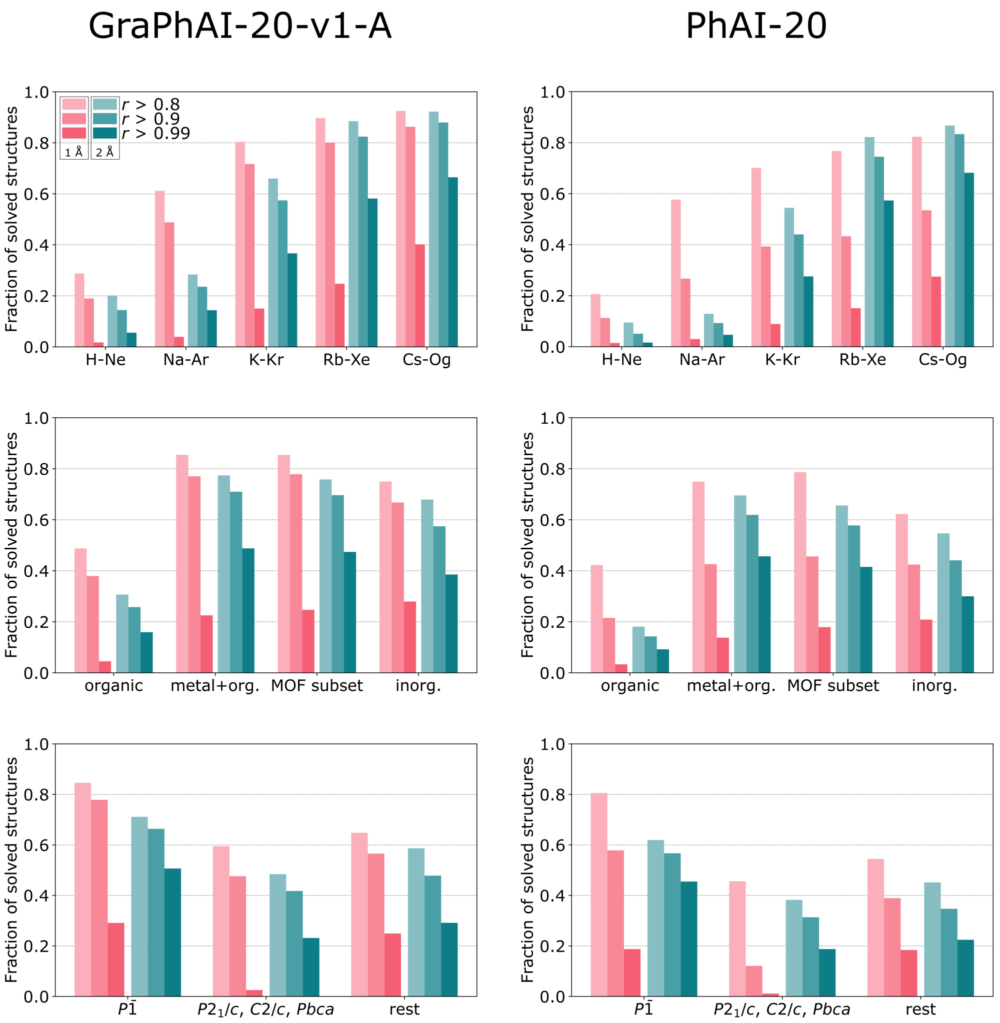

Fig. S5 Results of phasing the test set of 10 000 centrosymmetric crystal structures with unit cell size up to 20 Å. Left: model GraPhAI-20-v1-A (GNN architecture). Right: PhAI-20 (equivalent CNN architecture). In each row, the data set is segregated by different criteria.

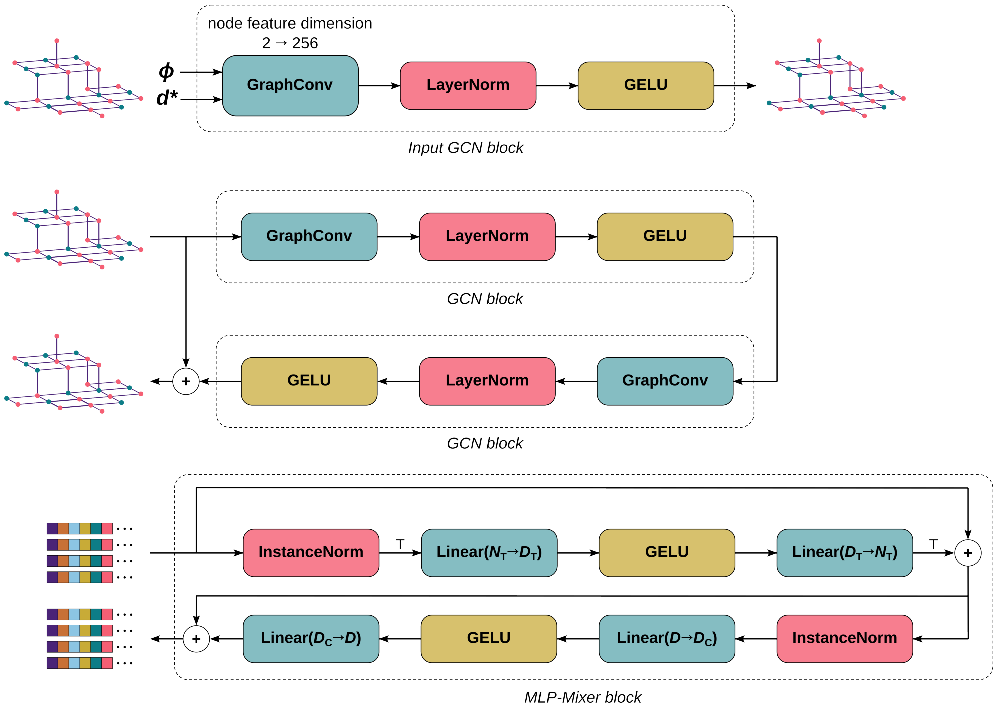

Fig. S6 Composition of GCN and MLP-Mixer blocks used in the GraPhAI-v1 architecture.

Hyperparameter	Value
Number of GCN blocks	9
Node embedding vector dimension	256
Number of MLP-Mixer blocks	8
Number of MLP tokens	256
MLP token dimension D	1024
MLP-Mixer dimension D_T	512
MLP-Mixer dimension D_C	2048

Table S1 Architectural hyperparameters of GraPhAI-v1 models.

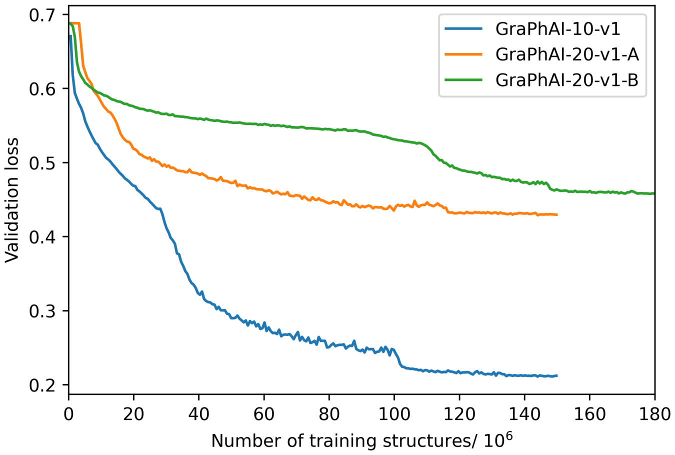

Fig. S7 Validation loss (weighted sum of binary cross-entropy per reflection) over the course of training for the three described GraPhAI-v1 models.
